# Hospital Meetings, &c.

**Published:** 1899-04-08

**Authors:** 


### The Institutional Workshop.

### HOSPITAL MEETINGS, &C.

### HOME HOSPITALS ASSOCIATION FOR PAYING PATIENTS.

Sir Henry Burdett presided at the annual general meeting of the Home Hospitals Association on Thursday, March 23rd, at Fitzroy House, Fitzroy Square.

The Hon. Secretary (Mr. T. Almond Hind) read the report, which showed that the hospital had afforded accommodation to an increasing number of patients, and that its popularity among the medical profession showed no sign of decrease. The total numbers admitted during the year were: Patients, 313; friends, 40; 73 applications having had to be refused for various reasons. The average stay of each patient was a little over 15 days, the average number in residence during the year being 14½. Fifty-eight different medical men had cases in the hospital during the year, and these all expressed themselves well satisfied with the arrangements and nursing of the establishment.

The association is not, of course, worked for profit, but the accounts show its finances to be in a satisfactory condition. The total receipts during the year were £5,326, and the total expenditure £5,365, increases as compared with last year of £119 and £139 respectively. The increase in expenditure was chiefly due to legal expenses. The death of the Duke of Northumberland, the president of the association, was referred to with regret, as were also those of Sir Richard Quain, Dr. C. J. Hare, and Mr. Hudson, the solicitor. The last-named gentleman's office had been filled by Mr. R. Turnbull.

The Chairman, in moving the adoption of this report, observed that the association might now be considered to have arrived at maturity, since this was their twenty-first meeting. He proposed to take a glance backward to the time when they started, and at the effect of what had happened since then, but he could not begin without a reference to those who had left them. He thought it showed that they had received the support of those who had reached a mature and experienced age when he recalled the fact that of all those who took a prominent part at the crowded Mansion House meeting at which the association was started, either for or against the idea, only three survivors remained in addition to himself, and of these, one, a supporter of the project from the first, was present with them that day. They could not but deeply lament the death of their president, the Duke of Northumberland. From the very outset he was an absolute believer in the principle for which they stood, and had always supported it in the most liberal way, as well as all schemes for the promotion of the welfare of the poorer classes in London. He himself felt that death as a great personal loss. Sir Richard Quain's death also was a loss to them; he had given them his assistance from the commencement down to the last day when he was able to attend a meeting of the Board, and no one had done more for the association. Dr. Hare, again, was an early supporter. They would recall how he had benefited by the establishment in a peculiar way, and so had come to be one of their firm friends. When, after some consideration and delay, they finally selected Berkeley House in Manchester Square, and bought it from Mr. Galsworthy for a large sum, they found themselves confronted by the opposition of the inhabitants of the square. These people took action on a clause in the lease, and although Sir George Jessel did all he could to prevent it, they were ultimately ejected. Then came Dr. Hare's opportunity. He had always had an eye on Berkeley House, and he was glad to come forward and take it of them; so that they got out of a great difficulty in having the house on their hands, and were able to make Dr. Hare happy, and make a friend of him at the same time. In Mr. Hudson, their solicitor, too, they had lost a useful friend. He felt that here a few words of remembrance were due to those who had done yeoman service in the cause they had at heart, and on such an occasion it was not inappropriate to include also Mr. John Walter, of the *Times*. The association was largely the creation of the metropolitan Press, which had always warmly supported them. A leading article appeared in the *Times* in which the question was asked—Why were these hospitals both for adults and children among the poor, and not for all classes? And this was followed by letters in the *Standard* of April, 1877, from which the actual scheme originated. Then came the meeting at the Mansion House, and since that time much of their progress had been due to the zeal and energy of their officers—Mr. Almond Hind, who had been hon. secretary for fifteen years; Mrs. Blewit (now Mrs. Almond Hind), who was with them for nine years, up to the time of her marriage, and whose record of service was one of the chief causes of their success; and since then, after an interregnum of two years, Miss Pearson, who had been lady superintendent for seven continuous years, and would, they hoped, be with them for many more. He could not leave the personal aspect of the subject without reference to their treasurer, Mr. Cox, who from the first had attended committee meetings and rendered immense service, with great devotion and sacrifice of time. He belonged to a type of man to whom Londoners ought to be very grateful, for they had a very great deal to do with the building up of all that made for the health and well-being of that vast community.

To come now to the progress of the association he was reminded that in 1890, when last he took the chair, there were but nine similar institutions to theirs; to-day there were no less than thirty-seven competing with them—all direct children of that, the parent association. In their first complete year in that building, 1881, they had 122 patients; in 1898, in spite of competition, they had 313. As an illustration of the progress that had been made in surgical science, it might be noted that while the usual stay of each patient was then about twenty-one days, it was now only about fifteen. This meant that recovery from operation—and most of their cases were such—was so much more rapid; and this was something they owed to the medical profession, and to Lord Lister especially. The number of friends staying with patients also showed a constant quantity; theirs was called a Home Hospital, and it was felt that patients ought to have the comfort and solace of the presence of their friends both before and after an operation.

Another gratifying feature was that the cost per patient had been materially reduced, for while it used to be as high as £18 it was now only about £14. This had been arrived at by close attention to managerial details and not by any curtailment of necessary expenditure; for efficiency and comfort there was still no place like Fitzroy House. There was no place more popular with medical men or where they would wish to send their patients or go themselves in preference. One of the main objects they had in view was to show that it was possible to conduct a hospital on a paying basis, where people could come and get the fullest benefits of the best hygienic system, of skilled nursing, and of those surroundings which make for health and speedy recovery. So far they had always been able to show a balance on the right side. This year, however, there was apparently a debit balance shown in the accounts, but that was explained by a cause which illustrated at once the efficiency of their ordinary arrangements and the risks to which such an institution must occasionally be exposed so long as it was human to err. One of their patients who had undergone an operation, and who was not very sensitive to pain, had been ordered to have hot bottles. One of these was, unfortunately, applied without the intervention of a piece of flannel, and as a consequence the friends sued the hospital, and the law expenses and compensation had cost them £282. Now, in their 21 years this was the first time anything of the kind had ever happened; the nurse responsible was a member of their staff in whom they had the completest confidence, and who found herself in the most distressful position of having forgotten something which she ought to have remembered, and which in an experience of 25 years she had never forgotten before. He offered her their sincerest sympathy, and he told her that though a most unfortunate occurrence, it was an accident which they were absolutely confident would never occur again, and which reflected no discredit on her and none on the institution. Turning from the immediate work of the association, the speaker desired to review the more general aspect of the origin and objects of the movement. It was first an educational movement. Speaking as one who had given much time and attention to the subject, he said deliberately that no hospital accommodation for any town or any country would be complete unless it provided for all classes of the community, and he would never rest content until such a change of system had been introduced as should give to everyone the opportunity of having the best hospital treatment in sickness or in accident, and, if they could afford it, of paying for it. It seemed absurd to complain of the abuse of hospitals and at the same time to declare that there was not enough room for cases requiring hospital treatment. Undoubtedly, the movement was making progress. In 1877, when that association was first suggested, there were only three small special hospitals which took in paying patients. To-day there were in the metropolitan area eight general hospitals and twenty-four special hospitals which did so. In 1896 the receipts from this source were 1·1 per cent., or £8,000; last year they amounted to 2 per cent., or £18,000. If they took all the hospitals in the country, and especially the Scotch hospitals, at least one in two of all provincial hospitals now admit paying patients; and taking the whole country the amount received in this way amounted to £50,000, or 2 per cent. of the whole income. He hoped to see the day when the accommodation of the hospitals would be so extended that no one who required it would be unable to get it. One remarkable sign of the spread of the idea he might mention. The Local Government Board had ordered that in their institutions all over the country accident and urgent surgical cases should be treated, and the cost recovered from the friends of the patient. He had no doubt whatever that ultimately the principle which that association represented would be successful. Another object they had in view was the removal of hospital abuse. The only effective way was to ascertain the cause and remove that cause. Patients everywhere, and medical men also, knew that if they wanted the best care and treatment the wisest course was to go straight to the nearest hospital. Therefore, they ought to address themselves to the removal of the cause of abuse by making adequate provision for all; for those who could afford to pay, and for those who could not. This could be done by having in every hospital a paying ward. He believed that to-day such a step would be welcomed by the whole medical profession, though twenty years ago many of them had regarded it with some dislike, provided proper arrangements were made. The safeguard for the medical man was simple—if the relief weie free the service should be free; if the relief were paid for they must also provide for the payment for the medical service. They already had the principle at work in cottage hospitals, where care was taken not only that the patients paid the hospitals, but that the medical profession should get their fees whenever it was possible. At the Great Northern Hospital, the latest example of a modern hospital designed as such an institution should be, it was provided in the estimates that they should have a department for paying patients. They determined that the pay wing should be a separate one, so as to secure that the outside practitioner could come in and follow his patient without interfering with the general administration. That was a great point. If they were to have successful pay wings to every hospital, as he hoped before long they would have, they must have a separate entrance so that they can be entered by the doctor without his having anything to do with the free portion of the institution. As an instance of the spread and development of the idea, the speaker quoted the case of John Hopkins, of Baltimore, U.S.A., who, in leaving funds for the building of a hospital there, declared that no trustees would adequately carry out his wishes unless they provided for all classes. In the deed of trust it was laid down that provision should be made for the indigent poor without charge and also for a limited number of patients who would pay for the treatment received and so enable a larger number of the poor to get the advantages of careful and skilful nursing and treatment.

If our hospitals were to be maintained as voluntary institutions—and God forbid that they should ever come on the rates—they would certainly have to adopt that course. No doubt one reason for hesitation on the part of the hospital managers was the fear that if they had a paying wing their supporters would say, "Oh! we want to give to the poor—not to those who can afford to pay." But the fact was that under the present system it could not be helped, to some extent at least. There were some cases so serious and so urgent that they must have hospital treatment—there was no way out of it. If supporters of hospitals once realised the true bearing of the facts they would refuse to give to any hospitals that had not a paying ward. If they would advocate this bridge between the pure paying hospital and the free hospital they would kill abuse; they would no longer have the present failure to provide accommodation in regard to the large class immediately above the indigent, and they would thus prove themselves worthy citizens, acting on the same lines as when they insisted that municipal government should be progressive and efficient. He was happy to know that owing to the movement in connection with the Prince of Wales's Fund, and to the bright prospects of the League of Mercy, the time was coming when they would be able to say to the hospitals generally: You are financed; you have the money; now look to the question of abuse, and deal with it intelligently as you are now in a position to do. And he was confident that with the introduction of paying wards hospital abuse would disappear. As to the willingness of the public to find the money, he had little doubt. If 21 years ago they could get £25,000 to prove the possibility of the principle of paying hospitals, they need not fear but that sufficient would be forthcoming when they appealed for sufficient to add paying wards to every institution, so as to be in a position to put a stop to the abuse of charity by those able to afford to pay for treatment, but who desired to get such treatment as hospitals alone could provide. Therefore, he said to hospital authorities everywhere, Look the question in the face with courage and intelligence, safeguarding every interest, and you will find that everybody will benefit—the poor, the labouring and the middle classes, and especially the medical profession—and we shall wonder why we delayed this noble work so long as we have done.

In conclusion, the speaker deprecated the putting of a weapon of attack into the hands of anti-vivisectors and other agitators by making it possible to say that money subscribed for the relief of the sick poor was spent on illegitimate objects, which they would do so long as any part went to the support of the medical schools, since these necessarily included bacteriological departments and assisted in physiological research. Those who entered for the law, the army, and the Church were all prepared to provide for their own education, and the medical profession was recruited from precisely the same classes. In London two things were wanting: one great medical school, and fees adequate to pay for the whole organisation necessary to make a young man fit for his profession. Parents were willing to pay, and it was bad business and wrong policy to have it otherwise. So there was one direction in which hospital authorities might cut down expenses—by refusing any longer to contribute towards the cost of medical education. That association was representing great principles, and as they started on their twenty-second year they might fairly hope soon to see such changes as would successfully place the voluntary system upon as sound a basis financially as it was morally and philanthropically.

Mr. T. Bryant seconded the resolution, and in supporting the chairman's advocacy of paying wards, instanced the success of a small institution with which he was connected—Bolingbroke Home Hospital, Wandsworth Common. There they took in cases at an average payment of two guineas a week—being whatever the patient's friends were prepared to pay—at the written request of a doctor in the neighbourhood; or in the case of an accident they wrote to the medical man, and, in conference between him and the house surgeon, arrangements were made as to what the patient was capable of paying. And most of them were willing and anxious to pay up to their power. He could not see why the same principle could not be applied to other hospitals, for it was certain that by this means a very large class would be able to get the benefits of hospital treatment for a remuneration far under what it would cost in their own homes. He endorsed Sir Henry's condemnation of the utilisation of hospital funds for the medical schools, but at the same time he held that such schools were absolutely necessary and must be maintained, not because they benefited the students, but for the good of the hospital itself. With a body of intelligent students watching and criticising and inquiring the reason for every action, it was impossible for the doctor to do anything but his very best for the patients.

On the motion of the Chairman, the present Duke of Northumberland was elected President of the association in succession to his father.

Mr. Mocatta, seconding Colonel Hamilton's motion for the re-election of the Working Committee, cordially supported the chairman's recommendation. He thought it was most desirable that hard-working, respectable people, who were neither paupers nor wealthy, should be able to have the manifest advantages of hospital treatment for their sick and injured without ruining themselves. He hoped soon to see a system of rebated payments instituted, and in every hospital a ward where such people could be admitted, and be able to leave the hospital not only benefited by their stay but with that sense of independence which was their right unimpaired.

Mr. Stoneham moved a vote of thanks to the executive staff. He had never met with better nursing, and he knew of no place where patients were better looked after.

On the motion of the Hon. Treasurer and Hon. Secretary, a cordial vote of thanks to the chairman concluded the meeting.

### NORTH LONDON HOSPITAL FOR CONSUMPTION.

The annual meeting of the governors of the North London Hospital for Consumption and Diseases of the Chest, Mount Vernon, Hampstead, N.W., was held on Wednesday, March 29th, at the secretary's offices and central out-patients' department, 41, Fitzroy Square, W., Mr. F. D. Mocatta presiding. The annual report of the committee of management stated that the death-rate was again low, viz., 4·8 per cent. The success of the "open-air" treatment of consumption, the report went on to say, has led the committee to consider the desirability of its extension at Hampstead, where the hospital was so well situated, where an abundance of pure fresh air can be obtained, and the structure adapted without great difficulty. At present two wards—one for men and the other for women—containing 24 beds, have been adapted for carrying out the treatment. The in-patient medical staff has been strengthened by the appointment of Dr. F. Rufenacht Walters, for many years senior out-patient physician at Fitzroy Square as a visiting physician to the hospital. Through the kindness of "two friends" the committee have been able to arrange for the supply of refreshments to the out-patients in the waiting-rooms at a very small fee. There was a substantial increase in annual subscriptions of £1,256, of which £1,000 was received from the Council of the Prince of Wales's Fund as an annual grant to enable the committee to open 15 additional beds. There is room in the hospital for 80 beds, and, thanks to this assistance, 75 are now occupied. Donations had increased to £3,306, of which £2,000 was received from the festival dinner. An anonymous donation of £3,000 had been received which is to endow two "asthma wards," and another donation of £1,000 endows a bed to be known as the "Alice bed"; £225 and £123 17s. were also obtained from the Hospital Sunday and Saturday Funds respectively, whilst the special building fund appeal realised £3,109. The total ordinary income was £6,869, and the extraordinary income £60, whilst the total ordinary expenditure amounted to £5,599, and the extraordinary expenditure to £378, giving a balance at December 31st last of £953.

The Chairman, in moving the adoption of the report, congratulated the meeting on the position disclosed, and alluded with satisfaction to the many good friends who had enabled the committee by their support to show a better balance-sheet than hitherto. For many years people had considered consumption an incurable disease, but that was found not to be so, and it promised by means of the open-air treatment to become in many cases a curable one; and it was pleasing to note that, notwithstanding they had to receive patients in advanced stages of the disease, their mortality amounted to under 5 per cent. It was also a source of much satisfaction that the work of the hospital was appreciated in high quarters, as shown by the £1,000 grant from the Prince of Wales's Fund, and by the other handsome donations. Sir Henry Harben was going to preside at the next festival dinner, and they hoped for great results from it. If they could only fill the remaining five beds, which they were always sorry to see empty, their satisfaction would be complete.

Mr. Benjamin A. Lyon, chairman of the committee of management, seconded the motion for the adoption of the report, which was unanimously agreed to.

The customary formal business having been transacted, the meeting concluded with a hearty vote of thanks to Mr. Mocatta for presiding.

### ROYAL EYE HOSPITAL.

The annual general meeting of the Royal Eye Hospital, Southwark, was held on Wednesday, March 29th ult., Professor Malcolm McHardy, the senior surgeon, presiding, in the absence of Sir Henry Irving, who was expected to take the chair. The report presented showed that the ordinary income amounted to £1,589, plus £900 from the banquet and £250 in legacies; the ordinary expenditure was £2,929, plus £104 for repairs. This worked out at a cost of less than 3s. 8d. per patient treated. The estimated cost of in-patients was £2 11s. 2d. each, and that of out-patients 2s. 4d. each—a very small amount for the results attained when it is remembered that prompt treatment not only averts blindness in many cases, but in most instances restores the bread-winner to the family. To keep the expenditure within the receipts the council were obliged to keep closed fourteen beds during most of the year. The meeting was held in one of the unoccupied wards as an object lesson in the needs of the institution. The Chairman, in moving the adoption of the report and accounts, called attention to the empty beds, and emphasised the economy with which the work of the hospital was carried on. The cost per occupied bed, he pointed out, was from 20s. to 21s. per week; 50 guineas per year. As evidence of the efficiency of the work done he mentioned that one of their consulting surgeons, Mr. C. Holthouse, F.R.C.S., had himself just been operated upon there for cataract, believing that nowhere could he get better service. The report was adopted, and the re-election of the council and cordial votes of thanks to the officers and staff agreed to. The Chairman announced that the annual festival dinner would be held at the Hotel Metropole on Friday, 21st instant, when Mr. W. M. Chinnery would preside. They were being warmly supported in the City, and hoped to raise sufficient to be able to utilise the vacant beds.

---

### ROYAL ORTHOPÆDIC HOSPITAL.

The chief result of the annual meeting of the Royal Orthopædic Hospital on Monday (March 27th) was the rejection by the governors present of seven members of the old committee of management, and the election of eight new members, with Mr. H. H. Marks, M.P., at their head, with the intention, so far as could be judged by what transpired, of vetoing altogether the proposal to remove the hospital from its present site (Hanover Square and Oxford Street). The resolution on the subject of which notice had been given was, however, not reached, owing to the protracted length of the proceedings. The committee were obviously greatly disconcerted and quite taken by surprise, while the opposition was evidently well organised, its proposals being carried with machine-like regularity, and by majorities of from thirty to forty votes in a packed meeting of about fifty. The management were so little prepared for such numbers that kitchen chairs had to be fetched in to provide seats, while the Press table was captured by youthful voters without sufficient *savoir faire* to make room for Press representatives, notes of the proceedings ha ving in conseqence to be taken standing.

Sir Walter Gilbey, the chairman of the committee, presided, supported by four members of the committee of twelve, most of the other members being stated to be ill or abroad.

The report stated that the committee had had under their grave consideration the present condition of the hospital, an old building, very ill-adapted for modern requirements; and had arrived at the conclusion that it was indispensable it should be rebuilt if the work of the charity was to be carried on properly. They expressed the opinion that it was desirable the hospital should be removed from its present site, and stated that a resolution to that effect would be submitted at the meeting.

There are between 500 and 600 governors of the hospital. Of these usually ten or a dozen at most attend the annual meetings. But on this occasion the board-room was filled to overflowing, there being about fifty persons present.

The report was adopted, the Chairman stating, in reply to a question, that this did not bind the meeting to agreement with the clauses respecting the necessity for removal.

The Chairman then proceeded to put the re-election of the committee *en bloc* according to custom, but this was objected to, and it was requested that the names should be taken seriatim. In answer to questions, the Chairman said there were only four of the committee present. He, however, warmly defended the way in which the committee had done their work; they met regularly week by week—a course adopted by very few hospitals in London—and it was very seldom there was any difficulty about forming a quorum. He could assure the governors that the business of the institution was as well managed and as well looked after as that of any commercial concern. They spent a great deal of time and attention about it. He for one, however, was not anxious to remain a member if they desired to replace him. He thought their better course would be to deal with the question of the site first, and elect the committee afterwards.

After some little discussion Sir Walter Gilbey's name as a member of the committee was put to the meeting and adopted unanimously, as was also that of Mr. Alderman Bell.

Mr. H. A. Reeves, honorary surgeon to the hospital, proposed the name of Mr. Harry H. Marks, M.P., as a member of the committee of management. This was seconded by Mr. Brodhurst, the senior honorary surgeon.

Mr. Bernard Parker supported Mr. Mark's candidature in a speech of considerable length. He was a personal friend of Mr. Marks, and should not venture to put him forwar unless he felt sure that he would be a great help to the institution. He went on to intimate that if it were decided to rebuild the hospital, Mr. Marks would be prepared to find £1,000 towards the building fund. The speaker then went into the state of the hospital's finances, pointing out how the deficit had grown and how the subscriptions and donations had fallen off of late years.

The Chairman again urged that the question of the site should first be dealt with. The present building was most unsuitable for a hospital and most insanitary. Mr. Clifford pointed out that the question before the meeting was the election of Mr. Marks, and asked for it to be put, whereupon the Chairman, saying he had not the least objection to any gentleman coming on the committee who wished to, put the resolution to the meeting, and it was carried unanimously.

The next name was that of Mr. Titus Barham, for whom seven votes were given, about forty voting against. Sir Ernest Clarke's name was next put, and he was unanimously elected. Then came that of Mr. Howard Colls, who, in spite of Sir Walter Gilbey's statement that he was one of the most regular attendants and a most useful supporter of the hospital in many ways, had only four hands held up in his favour and forty against.

The Chairman thereupon with some warmth tendered his resignation forthwith, being joined in this by Mr. Alderman Bell. They felt, Sir Walter said, in view of what had fallen from Mr. Parker, that this amounted to a vote of want of confidence.

Mr. Colls said he had for some years taken very considerable interest in the hospital and worked for it purely out of love. He should, however, be only too pleased to relinquish his seat, having many other calls upon his time and attention. He was quite sure, he said, that this adverse vote meant no personal animosity to himself, but was simply an expression of the very strong feeling there evidently existed among a section of the governors with regard to the site question, but of which, till then, the committee had known absolutely nothing. He saw now that that question should have been taken first, for nothing in the world would induce him to stay if it were decided to rebuild on the present site, and he thought it would be a simple scandal to spend £1,000, as they must at once if they were to remain there.

Mr. Reeves expressed himself as strongly opposed to the removal of the hospital from its present site. The meeting had been called, he said, by a notice which explicitly stated that a resolution on the subject would be submitted, and yet directly the question was raised the Chairman held a pistol to their heads by threatening to resign. The surgical staff and the committee had worked most harmoniously together, and personally he should be sorry to see Mr. Colls retire.

Attempts were then made to get Mr. Colls' name re-submitted, it being intimated that in view of the Chairman's attitude a different result might be expected, but neither Mr. Colls nor the Chairman would consent to this.

Mr. Drowers' name was then put and adopted unanimously, as was that of Mr. Studd; the other members of the old committee, Messrs. Gibson, Greenlees, Serena, Thornton, and Woulfe (hon. solicitor) being either not proposed at all or rejected by overwhelming majorities.

The old committee being thus disposed of by the election of five and the rejection of seven of its members,

Mr. Parker proposed Mr. James Head, chairman of the *Financial News*, who was seconded by Mr. Basil Dawson, and carried.

Mr. Parker was then proposed by Mr. Head, seconded by Mr. Miles, and carried, the hon. Solicitor, however, pointing out that no one could be elected a committeeman who was not already a governor, but being assured by Mr. Parker that Mr. Head was duly qualified.

Mr. Clifford proposed Mr. Frank Matthews, who was seconded by Mr. Basil Dawson, and elected.

Mr. W. Gordon Campbell was proposed by Mr. Barry, seconded by Mr. Toombs, and elected.

Major Ricarde-Seaver was proposed by Mr. Head, seconded by Mr. Parker, and elected.

Mr. T. Clifford was proposed by Mr. Reeves, seconded by Mr. Baker, and elected.

Mr. Reeves was proposed by Mr. Matthews, seconded by Mr. Pinner, and elected.

After the re-election of the trustees, treasurer, and auditors without discussion, that of the medical staff came up, when Mr. Clifford proposed their election for life.

Mr. Woulfe, the hon. solicitor, thought that under Law No. 7 it was incompetent for the medical staff to be elected for longer than one year at a time, and eventually the staff were re-elected for the ensuing year, the matter of their life election to be considered by the new committee.

The resolution as to the removal of the hospital was the next business.

Mr. Colls, in view of the lateness of the hour, moved that the committee be requested to take into consideration the question of removal or rebuilding of the hospital on its present site, and report to a general meeting to be summoned for the purpose. It transpired that an offer of £28,000 had been made for the site, and this sum, it was thought, would be sufficient to purchase a site in a less valuable position, though near, and provide for the building as well.

Dr. Brodhurst thought they might get much more than £28,000, but that it would be better to stay where they were and enlarge and extend the premises.

After considerable discussion Mr. Coll's motion was carried, and the meeting separated with a vote of thanks to the chairman, having lasted nearly three hours.

